# Recent Modifications of Chitosan for Adsorption Applications: A Critical and Systematic Review

**DOI:** 10.3390/md13010312

**Published:** 2015-01-09

**Authors:** George Z. Kyzas, Dimitrios N. Bikiaris

**Affiliations:** Laboratory of Polymer Chemistry and Technology, Division of Chemical Technology, Department of Chemistry, Aristotle University of Thessaloniki, GR-541 24 Thessaloniki, Greece; E-Mail: dbic@chem.auth.gr

**Keywords:** chitosan, modification, grafting, cross-linking, adsorption, pollutants, dyes, metals, pharmaceuticals, capacity

## Abstract

Chitosan is considered to be one of the most promising and applicable materials in adsorption applications. The existence of amino and hydroxyl groups in its molecules contributes to many possible adsorption interactions between chitosan and pollutants (dyes, metals, ions, phenols, pharmaceuticals/drugs, pesticides, herbicides, *etc.*). These functional groups can help in establishing positions for modification. Based on the learning from previously published works in literature, researchers have achieved a modification of chitosan with a number of different functional groups. This work summarizes the published works of the last three years (2012–2014) regarding the modification reactions of chitosans (grafting, cross-linking, *etc.*) and their application to adsorption of different environmental pollutants (in liquid-phase).

## 1. Introduction

**Figure 1:**
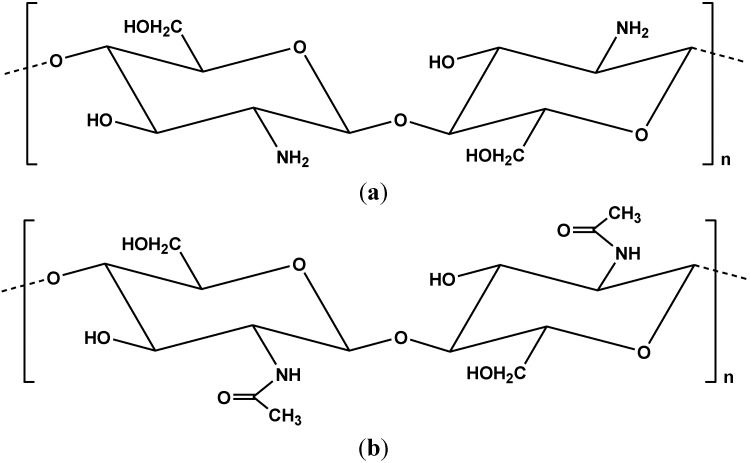
Chemical structure of (**a**) chitosan and (**b**) chitin.

Chitosan (poly-β-(1→4)-2-amino-2-deoxy-d-glucose) is a nitrogenous (amino-based) polysaccharide (Figure 1a), which is produced in large quantities by *N*-deacetylation of (its origin compound) chitin [1,2,3]. Chitin (poly-β-(1→4)-*N*-acetyl-d-glucosamine) can be characterized as one of the most abundant natural biopolymers (Figure 1b) [4,5]. Chitin exists in marine media and especially in the exoskeleton of crustaceans, or cartilages of mollusks, cuticles of insects and cell walls of micro-organisms. Chitosan can be easily characterized as a promising material not only due to its physical properties (macromolecular structure, non-toxicity, biocompatibility, biodegradability, low-cost, *etc.*) [2], and applications to many fields (biotechnology, medicine, membranes, cosmetics, food industry, *etc.* [6,7,8,9,10,11,12,13,14,15,16,17,18]), but also its adsorption potential.

Since the primary research work of Muzzarelli in 1969, who described the synthesis and adsorption evaluation of chitosan for the removal of metal ions from organic and sea waters [19], numerous papers have been published regarding the use of chitosan as adsorbent for decontamination of wastewaters (or effluents, sea waters, drinking samples, *etc.*) from various pollutants, either organic (dyes, phenolic and pharmaceutical compounds, herbicides, pesticides, drugs, *etc.*) or inorganic species (metals, ions, *etc.*). In order to obtain a more realistic view of the published works regarding the adsorption use of chitosan during time-periods, the following results were exported after the Scopus database screening (using the terms “chitosan” in combination with “adsorption” or “removal”): (i) 15 papers for 1969–1990; (ii) 116 papers for 1991–2000; (iii) 811 papers for 2001–2010, and (iv) 850 papers for 2011–2014. The results are relative and approximate, because many other papers are not present in the Scopus database or the search is not accurate. However, in any case, the trend is clear.

As it is clearly understood, the major advantage of chitosan is the existence of modifiable positions in its chemical structure. The modification of chitosan molecule with (i) grafting (insert functional groups) or (ii) cross-linking reactions (unite the macromolecular chains each other) leads to the formation of chitosan derivatives with superior properties (enhancement of adsorption capacity and resistance in extreme media conditions, respectively). In the case of grafting reactions, the addition of extra functional groups onto chitosan increases the number of adsorption sites and consequently the adsorption capacity. On the other hand, the cross-linking reactions slightly decrease the adsorption capacity because some functional groups of chitosan (*i.e.*, amino or hydroxyl groups) are bound with the cross-linker and cannot interact with the pollutant. As a general comment, in more recent years (after 1990), researchers have attempted to prepare chitosan-based adsorbent materials modifying the molecules of chitosan.

The scope of this work is to gather and summarize the most recent and updated published works of the last three years (2012–2014) regarding the modification reactions of chitosan derivatives (grafting, cross-linking, *etc.*). However, the present work is related to only chitosan modified materials synthesized for adsorption application (to different environmental pollutants) and not the sum of modified chitosan materials used for other applications (food science, membrane technologies, *etc.*). Scopus database exports 368 review articles for the wide term “chitosan”, but only 10 for the combination “chitosan” and “adsorption” or “removal” [20,21,22,23,24,25,26,27,28,29]. Therefore, there is a gap in updating the literature published in the last three years emphasizing the modified chitosan adsorbents.

## 2. Modified Chitosans for Dye Adsorption

Auta and Hameed prepared a composite of chitosan and clay for both batch and fixed-bed adsorption experiments [30]. Modified Ball clay (MBC) and chitosan composite (MBC-CH) were the adsorbents used for removal of Methylene blue (MB) from aqueous solutions. For the synthesis of MBC-CH, chitosan was dissolved in acetic acid in order to be mixed and then an amount of MBC was added and stirred for 1 day. The resulted mixture was dropped to NaOH solution in order to form beads. After that, a procedure of freeze-drying was carried out (particle size between 0.5 and 2.0 mm). The effect of pH was tested in the range of 4–10, while the optimum value found was at alkaline conditions. The isotherm curves were fitted to the Langmuir, Freundlich and Redlich-Peterson models with their non-linear expressions (Q_m_ = 259.8 mg/g at 30 °C). In the present study, the abbreviation of Q_m_ corresponds to the maximum theoretical adsorption capacity calculated after fitting to the Langmuir (or Langmuir-Freundlich in some cases) equation. The adsorption columns presented equilibrium capacities (for C_0_ = 200 mg/L; bed depth = 3.6 cm; flow rate = 5 mL/min) equal to 70 mg/g for MBC and 142 mg/g for MBC-CH.

The same dye (MB) was studied by another research group preparing a magnetic nanocomposite of chitosan/β-cyclodextrin (CDCM) [31]. For its preparation, maleoyl-β-CD was prepared following the method of Binello *et al.* [32]. After SEM (scanning electron microscopy) studies, the shape of the majority of CSCM particles was found to be spherical (~100 nm). The effect-of-pH experiments demonstrated that the highest MB removal was in the range of 4–6 (more protons were available to protonate amino groups to form NH_3_^+^ because the pka of chitosan is 6.0–7.0). The Q_m_ after fitting (Langmuir, Freundlich equations) was estimated to be 2.78 g/g (30 °C).

**Figure 2:**
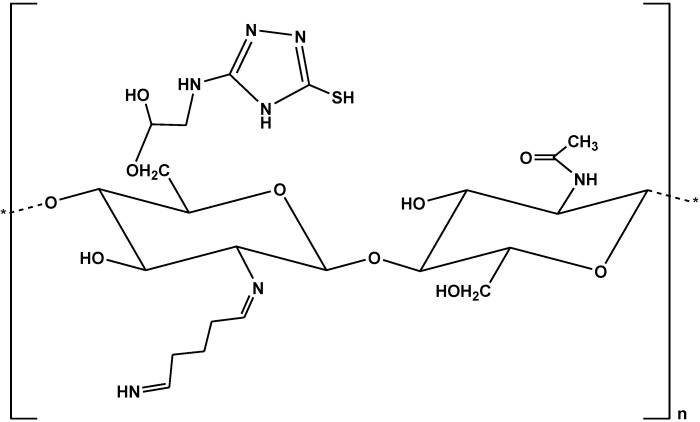
Chemical structure of the modified chitosan resin [33].

A study of Elwakeel *et al.* describes the preparation of a resin based on chitosan and glutaraldehyde [33]. The chemical modification was chemically achieved using NH_4_OH for producing the resin, which was then cross-linked with epichlorohydrine. This reaction took place between hydroxyl groups of chitosan molecules. The resultant resin was further modified with 3-amino-1,2,4 triazole,5-thiol to synthesize the final resin (Figure 2). The resin was prepared for binding/removing a cationic dye (BBR250) from aqueous media.

The surface area was estimated to be 371.1 m^2^/g, while the water regain was 17%. The experimental data were fitted to Langmuir and Freundlich model and the adsorption capacity (Q_m_) was found to be 0.8 mmol/g at 25 °C.

**Figure 3:**
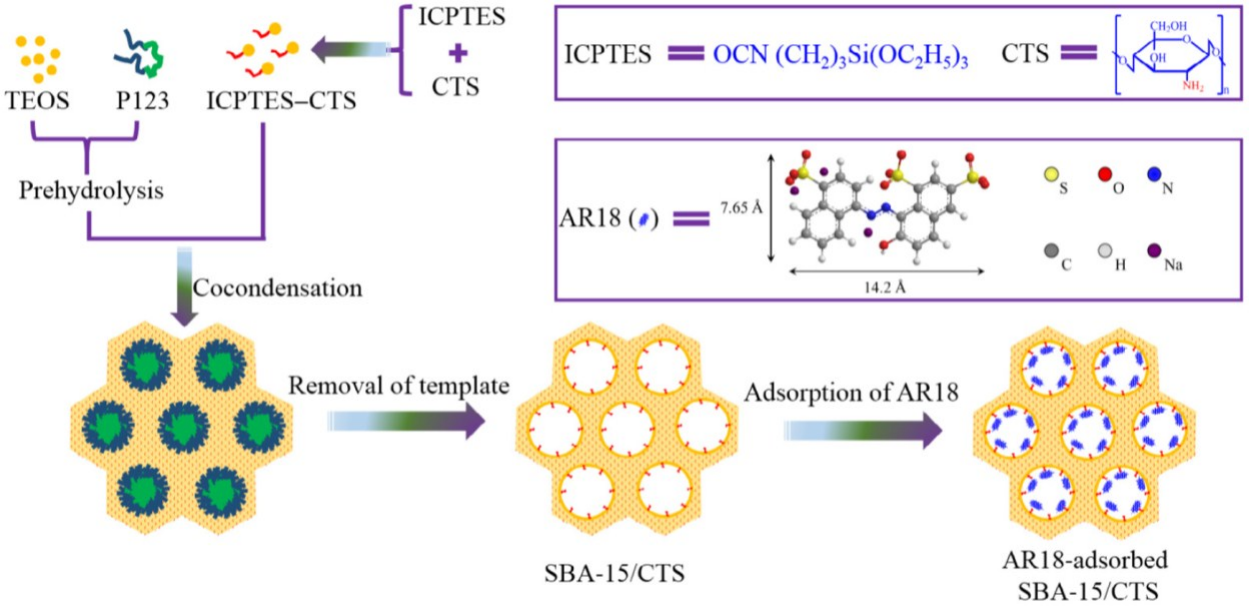
Preparation scheme of SBA-15/CTS and its application for the adsorption of AR18. Reprinted with permission from [34], Copyright © 2014 Elsevier Inc.

An anionic dye (AR18) was selected to investigate the adsorption properties of chitosan-functionalized with siliceous mesoporous SBA-15 [34]. The final products were SBA-15/CTS(5%), SBA-15/CTS(10%), and SBA-15/CTS(20%). CTS-modified SBA-15 composites were prepared according to rehydrolysis-condensation strategy (Figure 3).

After characterization, it was observed that the pore sizes of SBA-15, SBA-15/CTS(5%), SBA-15/CTS(10%), and SBA-15/CTS(20%) are 6.6, 6.5, 6.6, and 6.7 nm, respectively, with narrow distributions. The latter demonstrated that the porosity of those materials was not affected by the incorporation of chitosan due to condensation. CTS-modified SBA-15 composites were hydrophilic and the pH-effect experiments (in the range of 4–7) showed a strong dependence of dye uptake on pH (higher adsorption at low pH values; optimum pH value was 2). After fitting with Langmuir model, the highest Q_m_ was 201.2 mg/g at 30 °C for SBA-15/CTS(20%).

**Figure 4:**
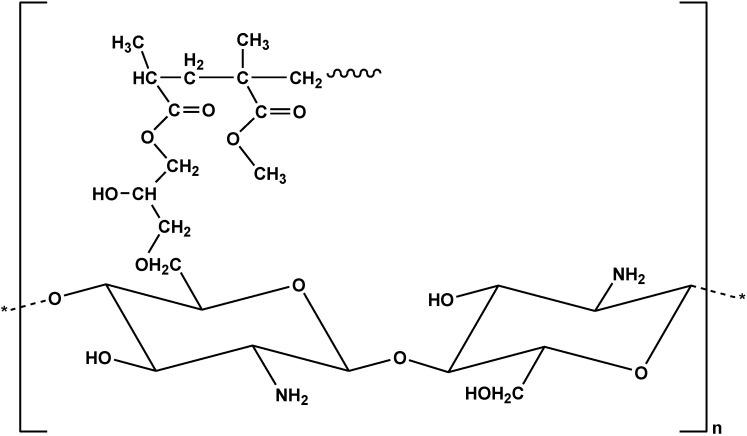
Chemical structure of chitosan grafted with poly(methyl methacrylate).

Another anionic dye (RB19) was successfully removed using chitosan grafted with poly(methyl methacrylate) (Figure 4) [35]. The synthesis included two steps: (i) functionalization of chitosan (CTS) with glycidyl methacrylate (GMA) in aqueous solution with pH = 3.8; and (ii) synthesis of the copolymer-(CTS-GMA)-g-PMMA using the mixed solvent of acetic acid and tetrahydrofuran (v/v, 2/1). The molar ratio of CTS/GMA/MMA was 1/1/1. The maximum dye uptake was 1498 mg/g (Langmuir fitting) at 30 °C (pH = 3).

Guo *et al.* modified the chitosan molecule using bentonite [36]. Chitosan modified bentonite (CTS-Bent) and chitosan-hexadecyl trimethyl ammonium bromide modified bentonite (CTS-CTAB-Bent) were the two final products after modifications and were tested for the removal of WASC dye from aqueous solutions. For the synthesis of the materials, a fixed amount of bentonite was added into deionized water and then quaternary ammonium salt solution was added to the above mixture with strong stirring. Chitosan solution was added step by step at a certain temperature. After stirring and cooling down to room temperature, the sample was filtered and washed with deionized water until no bromide ion detected by silver nitrate solution. From the FTIR spectra, a new band at 1564 cm^−1^ was related to the NH_2_ vibration mode of the chitosan, which indicated that the chitosan molecule was inserted into the interlayer space of the bentonite. The optimum synthesis conditions for the adsorption of 100 mg/L WASC solution were 1% chitosan and 10% CTAB at 80 °C and 2.5 h. So, the final removal for 1CTS-10CTAB-Bent was higher than 85% (Q_m_ = 102 mg/g) [36].

A more complex modification was recently published by Li *et al.* aiming to remove lysozyme using magnetic chitosan, which was previously modified with an affinity dye-ligand (RR120) [37]. The effect of this modification was determined by the increased adsorption amount of lysozyme (116.9 mg/g) on dye-modified microspheres, compared to only absorbing 24.6 mg/g on unmodified magnetic chitosan microspheres. The dye was grafted with covalent bonds onto the surface of magnetic chitosan microspheres via a nucleophilic substitution reaction.

Nezic *et al.* published a research article which presented data about synthesis of chitosan/zeolite composite [38]. In particular, chitosan/zeolite A films were produced after mixing chitosan solutions and dispersions of zeolite A in water. The zeolite A amount in the films was determined to be 40% of chitosan mass. The final films had no color and their thickness was approximately 0.1. The surface of the films was uniform with many agglomerates which were equally distributed all over the surface. An anionic dye used for adsorption evaluation (BO16); the equilibrium data were fitted to Langmuir and Freundlich models, presenting Q_m_ = 305.8 mg/g. This value was taken at pH = 6, which preliminarily was found to be optimum (after pH-effect experiments in the range of 4.0–7.5).

Chitosan-modified palygorskite (CTS-modified PA) was prepared by Peng *et al.* in order to remove an anionic dye (RY3RS) from aqueous media [39]. 3-Aminopropyl triethoxysilane (KH-550) was used as coupling reagent for the synthesis of the grafted derivative. The preparation route is simple; KH-550 was used to add aminopropyl groups onto the plygorskite (PA) surface. Briefly, PA (dried at 105 °C) was dispersed in fixed volume of toluene, and then KH-550 was added and dissolved (continuous stirring). The resulted solution was refluxed and PA was separated by filtration. The KH-550-modified PA was dried at room temperature in vacuum overnight. This product was soaked into glutaraldehyde; chitosan powder was dissolved in acetic acid solution. The two solutions were mixed and the reacted PA was separated using centrifugation and Na_2_CO_3_. CTS-modified PA was finally isolated by centrifugation, then washed with distilled water, and dried in vacuum [39]. For the adsorption experiments, the optimum pH value was found to be 4 and at this value the Q_m_ was 71.38 mg/g (Langmuir and Freundlich fittings).

Sadeghi-Kiakhani *et al.* synthesized a chitosan grafted polypropylene imine in dendrimer form [40]. As model dye compounds, two anionic dyes were used (RB5 and RR198). The adsorption capacities of the material for both dyes can be characterized as huge (RB5, 6250 mg/g; RR198, 5852 mg/g). The fitting was done with three models (Langmuir, Freundlich, Temkin).

Yan *et al.* studied a more complex adsorption phenomenon, in which two model dyes (AO7 and AG25) were competitively adsorbed onto beads of chitosan grafted with diethylenetriamine [41]. Langmuir and Freundlich equations were run for fitting. Q_m_ was calculated as 6.02 and 4.37 mmol/g for AO7 and AG25 in single-component solutions, respectively. For binary mixtures, 4.10 and 3.51 mmol/g were the respective values. From this study, characterization techniques do not exist.

Zhu *et al.* prepared chitosan-modified magnetic graphitized multi-walled carbon nanotubes (CS-m-GMCNTs) for removing CR from aqueous media [42]. The surface area of the prepared chitosan modified material was measured (BET analysis) as 39.20 m^2^/g. The maximum dye uptake was shown at pH = 6.3 and at this value the Q_m_ was calculated (Langmuir and Freundlich models) as 263.3 mg/g. Authors also made a direct comparison with other chitosan CR adsorbents of recent literature (not those published in 2012–2014); Q_m_ for CR adsorption on chitosan hydrobeads [43], chitosan [44], and *N*,*O*-carboxymethyl-chitosan/montmorillonite nanocomposite [45] were 92.59, 81.23, 74.24 mg/g, respectively.

A recent study of our research team [46] investigated the synthesis of a novel composite material (GO-Ch) consisting of cross-linked chitosan (Ch) and graphite oxide (GO) for the removal of RB5. All prepared products (GO, Ch, and GO-Ch) were ground to fine powders, with a size after sieving of 75–125 μm. The capacities found after fitting demonstrated that the functionalization of chitosan enhanced the Q_m_ (205, 224, and 277 mg/g (pH = 2) for GO, Ch, and GO-Ch).

**Figure 5:**
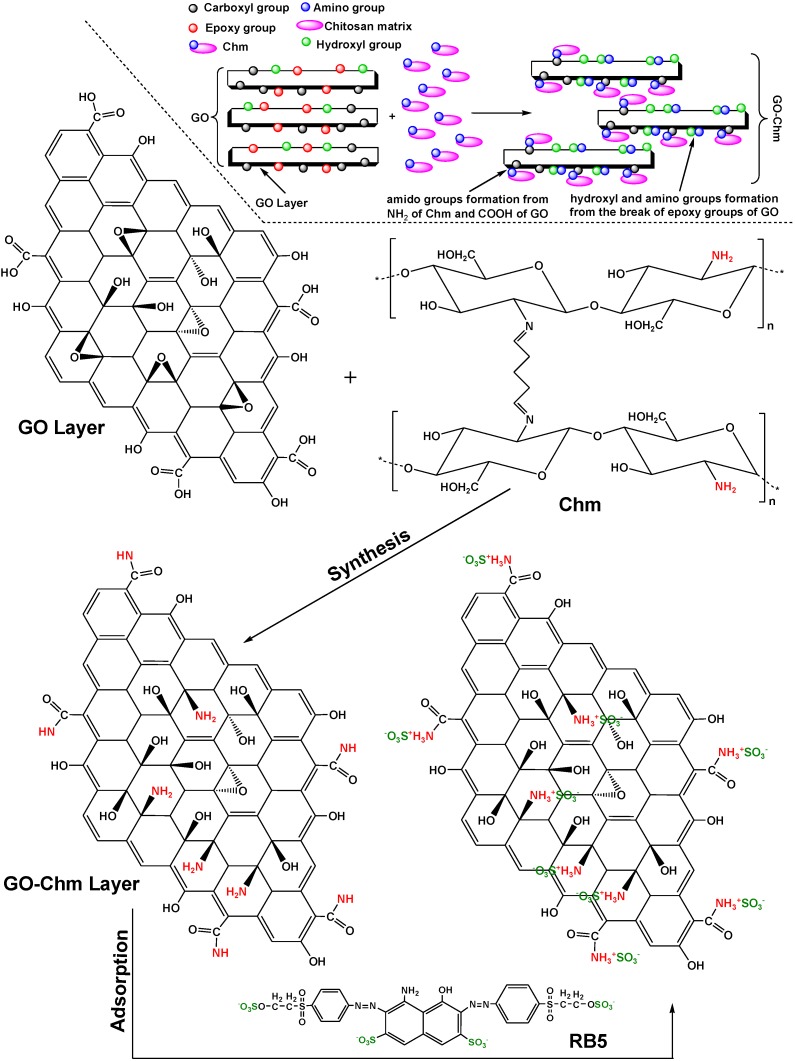
Proposed mechanism of synthesis of graphite oxide/magnetic chitosan composite (GO-Chm) after functionalization of magnetic chitosan (Chm) onto graphite oxide (GO). Proposed interactions of the Reactive black 5 (RB5) adsorption onto the prepared GO-Chm. Reprinted with permission from [47], Copyright © 2013 American Chemical Society.

Travlou *et al.* reveal the use of magnetic chitosan (Chm) instead of pure (Ch) in the functionalization and synthesis of graphite oxide/magnetic chitosan composite (GO-Chm) [47]. It was found that for GO, the Q_m_ was 221 mg/g (pH = 3) and 391 mg/g for GO-Chm. The possible interactions were illustrated in Figure 5 (found and proposed after characterization techniques).

Special note should be made of a selective adsorbent synthesized after modification of chitosan. Kyzas *et al.* prepared molecularly imprinted polymers of chitosan (CHI-MIPs) in order to selectively remove RR3BS (anionic dye) for aqueous solutions [48]. Q_m_ was approximately 35 mg/g and the selectivity very high (in a dye mixture containing other dyes).

Table 1 summarizes all the aforementioned modified chitosans and their respective adsorption application.

**Table 1:** Studies of the adsorption of dyes using modified chitosan adsorbents during 2012–2014.

Modified Chitosan Adsorbent	Dye	Isotherms	Q_m_	Ref.
Modified Ball clay/Chitosan composite	MB	L, F, R-P	259.8 mg/g	[30]
β-cyclodextrin/chitosan magnetic nanocomposite	MB	L, F	2788 mg/g	[31]
Chitosan/glutaraldehyde/3-amino-1,2,4 triazole,5-thiol resin	BBR250	L, F	0.8 mmol/g	[33]
Chitosan/SBA-15	AR18	L, F	201.2 mg/g	[34]
Chitosan grafted with poly(methyl methacrylate)	RB19	L	1498 mg/g	[35]
Chitosan modified bentonite	WASC	H, L, F, T, D-R	102 mg/g	[36]
Chitosan/CTAB modified bentonite	WASC	H, L, F, T, D-R	175 mg/g	[36]
Magnetic chitosan grafted with Reactive red 120	Lysozyme	L	116.9 mg/g	[37]
Chitosan/Zeolite A	BO16	L, F	305.8 mg/g	[38]
Chitosan-modified palygorskite	RY3RS	L, F	71.38 mg/g	[39]
Chitosan grafted with polypropylene imine	RB5	L, F, T	6250 mg/g	[40]
	RR198	L, F, T	5855 mg/g	[40]
Chitosan grafted with diethylenetriamine	AO7	L, F	6.02 mmol/g	[41]
	AG25	L, F	4.37 mmol/g	[41]
Chitosan-modified magnetic graphitized multi-walled carbon nanotubes	CR	L, F	263.3 mg/g	[42]
Chitosan/Graphite oxide composite	RB5	L-F	277 mg/g	[46]
Magnetic chitosan/Graphite oxide nanocomposite	RB5	L-F	391 mg/g	[47]
Chitosan-Molecularly Imprinted Polymers	RR3BS	L, F	35 mg/g	[48]

L: Langmuir; F: Freundlich; H: Henry; T: Temkin; D-R: Dubinin-Radushkevich; L-F: Langmuir-Freundlich; R-P: Redlich-Peterson.

## 3. Modified Chitosans for Metals/Ions Adsorption

The number of research articles published in literature during these three years regarding the use of chitosan adsorbents for metal/ion removal from aqueous solutions is by far higher than those for dyes removal. A possible explanation is that metals or/and ions cover a wider region of pollutants than dyes which are more toxic. Another reason could be a particular adsorption mechanism dominant in the majority of chitosan-ions systems which is called chelation. Chelation refers to a fixed manner in which ions and molecules bind to metal ions. The International Union of Pure and Applied Chemistry (IUPAC) provides a detailed description of chelation [49]: it involves the creation or even the presence of two or more separate coordinate bonds between a polydentate (multiple bonded) ligand and a single central atom [49]. In the majority of cases, the ligands are organic-based compounds (namely chelants, chelators, chelating agents). Muzzarelli *et al.* have published many works describing the chelation of different ions with chitosan derivatives [50]. In the present work, the papers with metal/ion adsorption onto modified chitosan adsorbents will be described.

Arvand *et al.* prepared a modified with 3,4-dimethoxybenzaldehyde chitosan derivative (Chi/DMB) for the removal and determination of Cd(II) from waters [51]. The successful synthesis of the new material was confirmed with FTIR spectrum before and after modification. Two new bands were recorded in Chi/DMB; A band at 1655 cm^−1^ can be attributed to an imine bond (N=C) and another one at 1562 cm^−1^ is associated with an ethylenic bond (C=C). The characteristic band of free aldehyde groups (1720 cm^−1^) disappeared. After pH-experiments, an increase of metal uptake was observed as the pH increased from 1.0 to 9.0. However, cadmium started to precipitate from solution and therefore the increased capacity at values higher than 7 may be due to a combination of both adsorption and precipitation on the surface. So, the value selected was 6.5. After running the Langmuir isotherm model, Q_m_ was found to be 217.4 mg/g.

The removal of U(VI) from wastewaters using chitosan-modified multiwalled carbon nanotubes (MWCNT-CS) was proposed by Chen *et al.* [52]. The maximum ion removal was at pH = 7. Using the Langmuir model, the maximum adsorption capacity was found to be 71 mg/g. Chen and Wang synthesized a xanthate-modified magnetic cross-linked chitosan (XMCS) for the removal of Co(II) [53]. The xanthate-modification was based on the treatment of cross-linked magnetic chitosan with NaOH solution and CS_2_. After stirring at room temperature (24 h) and washing with deionized water, the final derivative was dried at 70 °C. Langmuir and Freundlich isotherms calculated the Q_m_ for the material (18.5 mg/g).

Chethan and Vishalakshi attempted the selective modification of chitosan by incorporating ethylene-1,2-diamine molecule in a regioselective manner using *N*-phthaloylchitosan and chloro-6-deoxy *N*-phthaloylchitosan as precursors [54]. The derivatives that resulted were ethylene-1,2-diamine-6-deoxy-*N*-phthaloylchitosan (PtCtsEn) and ethylene-1,2-diamine-6-deoxy-chitosan (CtsEn) and were prepared for Cu(II), Pb(II) and Zn(II) removal. The capacities calculated (Langmuir model) for CtsEn were 41.6, 31.8, and 20.0 mg/g, for Cu(II), Pb(II), and Zn(II), respectively, while for PtCtsEn they were 32.3, 28.6 and 28.6 mg/g, respectively. Another study published by Debnath *et al.* presented the removal results of Cr(VI) after adsorption onto magnetically modified graphene oxide-chitosan composite [55]. The graphene oxide was prepared based on Hummers method [56], and the optimum pH found after adsorption experiments was 3.0. Langmuir, Freundlich and Redlich-Peterson models were used for fitting (Q_m_ ~75 mg/g, 25 °C).

Elwakeel *et al.* prepared a resin, which was a structure based on magnetic modified chitosan [57]. Chitosan was initially cross-linked with glutaraldehyde in magnetite presence and then chemically modified reacting with tetraethylenepentamine (TEPA). UO_2_(II) were removed presenting Q_m_ = 1.8 mmol/g (after fitting to Freundlich, Langmuir, and Dubinin-Radushkevich equations) at pH 4 (25 °C). In this study, column experiments were also carried out varying parameters (flow rate, *etc.*). Eser *et al.* chemically modified the raw chitosan with histidine (HIS-ECH-CB) for increasing the Ni(II) uptake [58]. Q_m_ was calculated with Langmuir (55.6 mg/g) and Freaundlich models. The cross-linking of material was done using epichlorohydrin, while the immobilization of histidine was performed after washing with Na_2_CO_3_ solution. The beads forms were placed in a recipient and 10% (w/v) histidine solution and added to a Na_2_CO_3_ solution. The mixture was stirred at 60 °C for 1 day and then the beads were washed until excess non-immobilized histidine was removed.

Gandhi *et al.* prepared a series of modified chitosan beads (CB) in order to remove Fe(III) from aqueous solutions [59]. However, the adsorption capacity of the modified forms of chitosan beads (protonated (PCB), carboxylated (CCB) and grafted CB (GCB)) was not high enough (7.042, 9.346, and 14.286 mg/g, respectively). On the other hand, a series of ions (Cu(II), Co(II), Zn(II), Hg(II), Pb(II)) were removed with another grafted chitosan derivative [60]. The modification reaction was based on the reaction of chitosan with 4,4′-diformyl-α-ω-diphenoxy-ethane. The maximum uptake was presented at pH = 5, and the adsorption capacities calculated were 12, 8, 12, 56, 50 mg/g for Cu(II), Co(II), Zn(II), Hg(II), Pb(II), respectively.

Li *et al.* formed an ethylenediamine modified yeast biomass after coating with magnetic chitosan (EYMC) [61]. Interesting morphological properties were exported after characterization (13.2 m^2^/g as the surface area), but the most interesting property was on the surface of the EYMC material. Numerous, small bumps existed on the surface, forming a large quantity of pores. These pores may significantly contribute to the transfer of Pb(II) ions to the surface of EYMC. After metal adsorption, the pores were adhered by Pb(II) [61]. The maximum uptake of Pb(II) was shown at pH = 4–6 and the Langmuir (both fitting with Freundlich) equation gave Q_m_ = 121.6 mg/g. The authors propose the following adsorption interactions between lead and chitosan derivative [61]:

(1) $$\begin{array}{l} \text{P}{\text{b}}^{2+}+\text{R}-\text{OH}+{\text{H}}_{2}\text{O}\to \text{R}-\text{OPb}-\text{OH}+2{\text{H}}_{3}{\text{O}}^{+}\\ \text{R}-\text{COOH}+\text{P}{\text{b}}^{2+}+{\text{H}}_{2}\text{O}\to {\left(\text{R}-\text{COO}\right)}_{2}\text{Pb}+{\text{H}}_{3}{\text{O}}^{+}\\ \text{R}-\text{N}{\text{H}}_{2}+\text{P}{\text{b}}^{2+}+{\text{H}}_{2}\text{O}\to -\text{N}{\text{H}}_{2}{\left(\text{PbOH}\right)}^{+}\\ \text{R}-\text{N}{{\text{H}}_{3}}^{+}+\text{PbC}{{\text{l}}_{3}}^{-}\to \text{R}-\text{N}{{\text{H}}_{3}}^{+}\text{PbC}{{\text{l}}_{3}}^{-} \end{array}$$

Thiocarbohydrazide-modified chitosan (TCHECS) was synthesized for removal of a series of ions (As(V), Ni(II), Cu(II), Cd(II), Pb(II)) [62]. The main application for this study was the evaluation of anti-corrosion ability of TCHECS and not adsorption. For this reason, Q_m_ was not calculated, but authors observed that the higher uptake was at pH = 9 with 55.6%–99.0% uptake. The same concept was kept in another study by the same research team and two new chitosan grafted materials were prepared [63] and tested just for the same ions (As(V), Ni(II), Cu(II), Cd(II), Pb(II)). Thiosemicarbazide (TSFCS) and thiocarbohydrazide (TCFCS) grafted chitosan were the adsorbents prepared, presenting 66.4%–99.9% and 71.5%–99.9% uptake for the two adsorbents, respectively.

Monier was targeted to the structural modification of chitosan, synthesizing chitosan-thioglyceraldehyde Schiff’s base cross-linked magnetic resin (CSTG) [64]. Mercury porosimetry data was used for the characterization of resin, and the average pore size was found to be 795 nm. BET analysis showed that the surface area was 70.5 m^2^/g. VSM plots indicated that the saturation of magnetization was 29.3 emu/g. This resin was used for the removal of three toxic metals (Hg(II), Cu(II, Zn(II)). The capacities of the resin for Hg(II), Cu(II, Zn(II) were estimated (Langmuir, Freundlich, Temkin equations) to be 98, 76, 52, mg/g, respectively. Another set of modified chitosan materials (chloroacetic grafted chitosan and glycine grafted chitosan) was produced after equivalent molar amounts of chitosan and glycine/chloroacetic acid in xylene solvent (130 °C for 3 h) [65] were obtained. The key factor of this procedure was the forced stop of this end reaction when the equivalent amount of water was obtained. Q_m_ was found to be 59.1 (Co(II)), 175.12 (Cu(II)) mg/g for chloroacetic grafted chitosan and 82.9, 165.91 mg/g for glycine grafted chitosan.

Rabelo *et al.* used only cross-linking reactions for modifying chitosan [66]. The agents used were glutaraldehyde (chitosan-GLA) and epichlorohydrin (chitosan-ECH). The adsorption experiments were done for Cu(II) and Hg(II) removal. Langmuir, Freundlich, Langmuir-Freundlich, Henry, and Toth isotherms fitted the equilibrium results. Chitosan-GLA presented Q_m_ equal to 2.8 and 3.3 mmol/g for Cu(II) and Hg(II) removal, respectively, while the values for chitosan-ECH were 2.3 and 3.5 mmol/g.

Gandhi and Meenakshi synthesized amino terminated hyperbranched dendritic polyamidoamine 1st generation (1ACB) chitosan beads after the grafting reaction onto the chitosan beads [67]. This modification was done in two stages: (i) Michael addition of methyl acrylate to amino groups on the surface and (ii) amidation of terminal ester groups by ethylene diamine. In this study, the modification reactions are more complex given the synthesis of both 2nd generation chitosan beads (2ACB) and 3rd generation (3ACB) ones. Those were prepared from 1ACB by repeating the above two processes. The authors suggest that for enhancing Cr(VI) adsorption, 3ACB should be further protonated and loaded with Zr(IV). Adsorption results for 3ACB gave Q_m_ equal to 224.2 mg/g.

**Figure 6:**
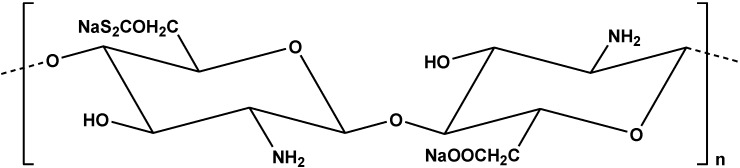
Structure of xanthate carboxymethyl grafted chitosan.

Repo *et al.* investigated the removal of Co(II) by EDTA-modified chitosan [68]. The material prepared has specific surface area of 0.71 m^2^/g and 1.8 × 10^−3^ cm^3^/g total pore volume (610 Å average pore size). This adsorbent tested for Co(II) removal and showed Q_m_ equal to 1.35 mmol/g (Langmuir, Sips equations) Song *et al.* prepared a novel xanthate carboxymethyl grafted chitosan derivative for removal of Cu(II) and Ni(II) from aqueous solutions (Figure 6) [69]. The effect of pH was tested in the range of 2.0–7.0 and Q_m_ was calculated using Langmuir and Freundlich models to be 174.2 mg/g and 128.4 mg/g for Cu(II) and Ni(II), respectively.

**Figure 7:**
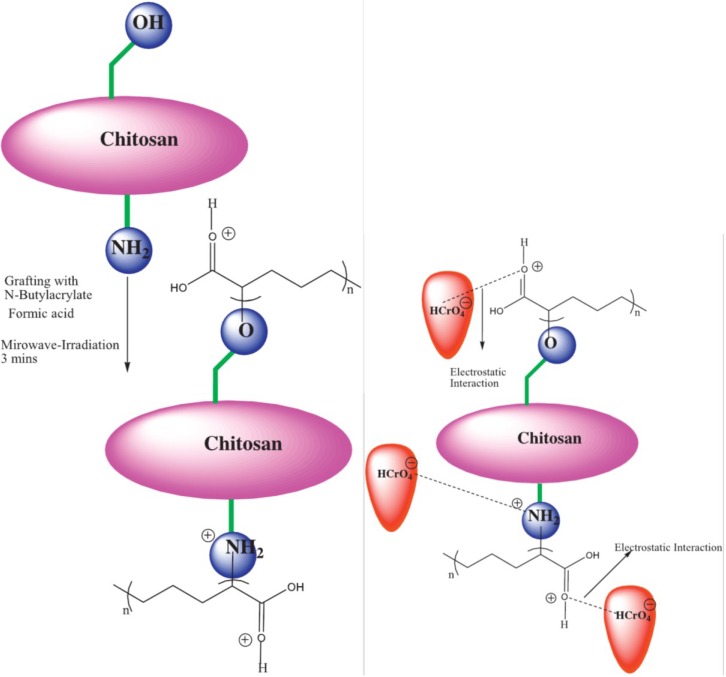
Interactions of chitosan, *N*-butylacrylate and chromium(VI). Reprinted with permission from [70], Copyright © 2014 Elsevier B.V.

The grafting of *n*-butylacrylate onto chitosan was studied for use as adsorbent in Cr(VI) uptake [70]. The synthesis procedure was assisted with microwave. FTIR spectrum confirmed the successful preparation of grafted derivative, presenting a peak at 1727 cm^−1^ (ester ‒C=O group) [70]. For the adsorption experiments, many isotherm models were tested (Langmuir, Freundlich, Dubinin-Radushkevich, Temkin, Elovich and Redlich) and Q_m_ was found 17.15 mg/g [70]. The authors illustrate a useful scheme for the main interactions (Figure 7).

**Figure 8:**
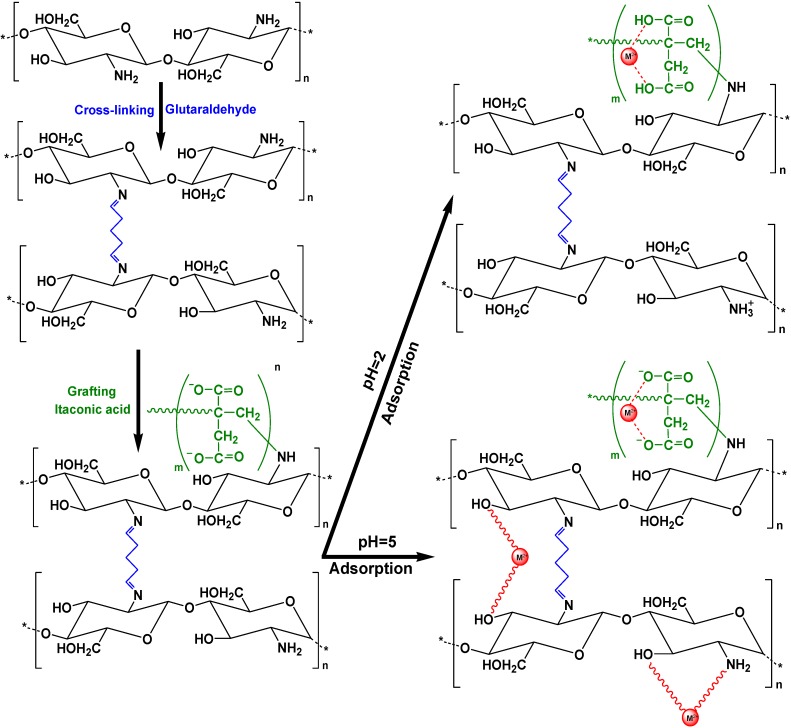
Cross-linked and grafted chitosan (CS) with itaconic acid (IA) and interactions between the material cross-linked with glutaraldehyde (CS-g-IA(G)) and metal ions. Reprinted with permission from [77], Copyright © 2014 American Chemical Society.

Suc and Ly achieved the preparation of chitosan flakes which were modified with citric acid and cross-linked with glutaraldehyde [71]. This material was applied to Pb(II) removal, finding that Q_m_ was 101.7 mg/g (Langmuir, Freundlich, Temkin and Dubinin-Radushkevich models). The authors ran their adsorption experiments at pH = 5 in order to avoid precipitation of Pb(OH)_2_. Montmorillonite modified with chitosan (CTS-MMT) was another modification achieved by Wang *et al.* for the removal of only Co(II) [72]. 150 mg/g was the Q_m_ calculated (Langmuir, Freundlich and Temkin equations). Moreover, triethylene-tetramine modified magnetic chitosan (TETA-MCS) was prepared for the removal of Th(IV) from aqueous solutions [73]. Similarly, Langmuir, Freundlich and Temkin models were run for calculation of the maximum adsorption capacity (133.3 mg/g at 25 °C). The same team prepared a similar modified derivative (diethylenetriamine-functionalized magnetic chitosan) for U(VI) removal (65.16 mg/g at 25 °C) [74]. In addition, Yang *et al.* modified magnetic chitosan using α-ketoglutaric acid (α-KA-Fe_3_O_4_/CS) for removal of Cd(II) from aqueous solution (Q_m_ = 201.2 mg/g) [75]. Magnetic chitosan was also prepared by Kyzas and Deliyanni for Hg(II) removal [76]. The optimum pH value for adsorption was 5 and Q_m_ (fitting with Langmuir and Freundlich model) was 152 mg/g. Furthermore, Kyzas *et al.* used two other modified chitosan adsorbents which were grafted with itaconic acid (CS-g-IA) and cross-linked either with glutaraldehyde (CS-g-IA(G)) or epichlorohydrin (CS-g-IA(E)) (Figure 8). These adsorbents were tested for Cd(II) or Pb(II) uptake. The authors demonstrated that Q_m_ (Cd(II)) was 405 mg/g and 331 mg/g for CS-g-IA(G) and CS-g-IA(E), respectively, (Q_m_ were equal to 124 and 92 mg/g before grafting, respectively) [77].

Table 2 summarizes the aforementioned modified chitosans and their respective adsorption applications to metals/ions.

**Table 2:** Studies of the adsorption of metals/ions using modified chitosan adsorbents during 2012–2014.

Modified Chitosan Adsorbent	Metals/Ions	Isotherms	Q_m_	Ref.
Chitosan grafted with with 3,4-dimethoxybenzaldehyde	Cd(II)	L	217.4 mg/g	[51]
Chitosan modified multiwalled carbon nanotubes	U(VI)	L	71 mg/g	[52]
Xanthate-modified magnetic cross-linked chitosan	Co(II)	L, F	18.5 mg/g	[53]
Ethylene-1,2-diamine-6-deoxy-chitosan	Cu(II), Pb(II), Zn(II)	L	41.6, 31.8, 20.0 mg/g	[54]
Ethylene-1,2-diamine-6-deoxy-*N*-phthaloylchitosan	Cu(II), Pb(II), Zn(II)	L	32.3, 28.6, 18.6 mg/g	[54]
Magnetically modified graphene oxide-chitosan composite	Cr(VI)	L, F, R-P	82 mg/g	[55]
Magnetic cross-linked chitosan grafted with tetraethylenepentamine	UO_2_(II)	L, F, D-R	486 mg/g	[57]
Cross-linked chitosan modified with histidine	Ni(II)	L, F	55.6 mg/g	[58]
Protonated chitosan beads	Fe(III)	L, F	7.042 mg/g	[59]
Carboxymethyl chitosan beads	Fe(III)	L, F	9.346 mg/g	[59]
Grafted chtiosan beads	Fe(III)	L, F, T	14.286 mg/g	[59]
Chitosan grafted with 4,4′-diformyl-α-ω-diphenoxy-ethane	Cu(II), Co(II), Zn(II),	L	12, 8, 12 mg/g	[60]
Chitosan grafted with 4,4′-diformyl-α-ω-diphenoxy-ethane	Hg(II), Pb(II)	L	56, 50 mg/g	[60]
Ethylenediamine-modified yeast biomass coated with magnetic chitosan	Pb(II)	L, F	121.26 mg/g	[61]
Thiocarbohydrazide-modified chitosan	As(V), Ni(II), Cu(II)			[62]
	Cd(II), Pb(II)			[62]
Thiosemicarbazide grafted chitosan	As(V), Ni(II), Cu(II)			[63]
	Cd(II), Pb(II)			[63]
Thiocarbohydrazide grafted chitosan	As(V), Ni(II), Cu(II)			[63]
	Cd(II), Pb(II)			[63]
Chitosan-thioglyceraldehyde Schiff’s base cross-linked magnetic resin	Hg(II), Cu(II, Zn(II)	L, F, T	98, 76, 52 mg/g	[64]
Chloroacetic grafted chitosan	Co(II), Cu(II)	L	59.1, 175.12 mg/g	[65]
Glycine grafted chitosan	Co(II), Cu(II)	L	82.9, 165.91 mg/g	[65]
Chitosan cross-linked with glutaraldehyde	Cu(II), Hg(II)	L, F, L-F, H	177.8, 661.5 mg/g	[66]
Chitosan cross-linked with epichlorohydrin	Cu(II), Hg(II)	L, F, L-F, H	146.1, 681.7 mg/g	[66]
Amino terminated hyperbranched dendritic polyamidoamine 3rd generation chitosan beads	Cr(VI)	L, F, D-R	224.2 mg/g	[67]
EDTA-modified chitosan	Co(II)	L, S	79.7 mg/g	[68]
Chitosan grafted with n-butylacrylate	Cr(VI)	L, F, D-R, T	17.15 mg/g	[70]
Xanthate carboxymethyl grafted chitosan	Cu(II), Ni(II)	L, F	174.2, 128.4 mg/g	[69]
Cross-linked chitosan with citric acid	Pb(II)	L, F, T, D-R	101.7 mg/g	[71]
Montmorillonite modified with chitosan	Co(II)	L, F, T	150 mg/g	[72]
Triethylene-tetramine modified magnetic chitosan	Th(IV)	L, F, T	133.3 mg/g	[73]
Diethylenetriamine-functionalized magnetic chitosan	U(VI)	L, F, S, D-R	65.16 mg/g	[74]
Magnetic chitosan grafted with α-ketoglutaric acid	Cd(II)	L, F, T	201.2 mg/g	[75]
Magnetic chitosan	Hg(II)	L, F	155 mg/g	[76]
Chitosan grafted with itaconic acid	Cd(II), Pb(II)	L-F	405, 334 mg/g	[77]

L: Langmuir; F: Freundlich; H: Henry; T: Temkin; D-R: Dubinin-Radushkevich; L-F: Langmuir-Freundlich; R-P: Redlich-Peterson; S: Sips.

## 4. Modified Chitosans for Other Species Adsorption

Apart from the main categories of pollutants (dyes, metals/ions), some other species were recorded in the literature regarding chitosan-modified adsorbents during the last three years. It is important to note that despite the fact there are not many papers on the subject, the under-removal of pollutants is toxic and hazardous for human health and environment; therefore, this subject deserves special attention. Furthermore, PAHs (polycyclic aromatic hydrocarbons) and PCBs (polychlorinated biphenyls) are indeed a serious class of contaminants. However, there were no published studies found in recent literature (as extensively studied in the present review article) regarding the use of chitosan as adsorbent for this type of contaminants.

Poon *et al.* studied the effect of different molar ratios of glutaraldehyde (cross-linkers) on the adsorption capacity of chitosan modified derivatives produced [78]. Three copolymers were synthesized beginning with the same mass of chitosan (6 g), but differentiating the volume of cross-linker in the mixing solution (3.6, 6.3 and 9.0 mL of glutaraldehyde (GLA) solution 50% (w/v) with density 1.106 g/L). Therefore, the calculated GLA/NH_2_ mole ratios were estimated to be 0.67, 1.17 and 1.68, respectively (samples denoted as CG-1, CG-2, and CG-3, respectively). For the adsorption evaluation, *p*-Nitrophenol was used a model compound, but classical types of isotherms were not exported/given. Only for particular initial phenol concentrations at three fixed values of pH (4.6, 6.6 and 9.0) were the respective adsorption capacities estimated. For CG-1, CG-2, and CG-3, the capacities were calculated as 0.285, 0.327, 0.381 mmol/g (at pH = 4.6). The respective values at pH = 6.6 were 0.289, 0.378, 0.436 mmol/g, while at pH = 9.0 they were 0.306, 0.451, 0.572 mmol/g, respectively. The authors concluded that increasing the cross-linker ratio would result in an increase of the absorption capacity. However, the maximum value occurs at the ration GLA/NH_2_. This is contrary to what has been published in numerous studies [77,79,80,81]. Authors also implied that for higher GLA ratio beyond the optimum found above, the result would be because of surface changes and inaccessibility of the sorption sites [78].

Magnetic chitosan derivatives were prepared for the removal of pharmaceutical compounds (diclofenac (DCF), clofibric acid (CA), carbamazepine (CBZ)) from aqueous media [82]. The magnetic chitosan had high sorption affinity for DCF and CA, but for CBZ no sorption was observed. The adsorption capacities of CA and DCF in the single-component solutions were 191.2 and 57.5 mg/g, respectively (Langmuir, Freundlich equations).

Chlorophenols (phenol, 2-chlorophenol (2-CP), 4-chlorophenol (4-CP), 2,4-dichlorophenol (DCP) and 2,4,6-trichlorophenol (TCP)) were also removed with adsorption onto cross-linked chitosan functionalized with β-cyclodextrin [83]. Q_m_ was calculated for phenol, 2-CP, 4-CP, DCP and TCP on CS-SA-CD and found to be 59.74, 70.52, 96.43, 315.46 and 375.94 mg/g, respectively.

Table 3 summarizes all the aforementioned modified chitosans and their respective adsorption application in other species.

**Table 3:** Studies of the adsorption of other species using modified chitosan adsorbents during 2012–2014.

Modified Chitosan Adsorbent	Pollutants	Isotherms	Q_m_	Ref.
Chitosan cross-linked with glutaraldehyde	*p*-nitrophenol			[78]
Magnetic chitosan	Diclofenac	L, F	191.2 mg/g	[82]
	Clofibric acid	L, F	57.5mg/g	[82]
	Carbamazepine	L, F	-	[82]
β-cyclodextrin/cross-linked chitosan	Phenol	L, F	59.74 mg/g	[83]
	2-chlorophenol	L, F	70.52 mg/g	[83]
	4-chlorophenol	L, F	96.43 mg/g	[83]
	2,4-dichlorophenol	L, F	315.46 mg/g	[83]
	2,4,6-trichlorophenol	L, F	375.94 mg/g	[83]
Chitosan grafted with sulfuric acid	Pramipexole	L-F	337 mg/g	[84]
Chitosan grafted with *N*-(2-carboxybenzyl)	Pramipexole	L-F	307 mg/g	[84]
Graphite oxide/Carboxyl-grafted chitosan	Dorzolamide	L-F	334 mg/g	[85]
Carboxyl-grafted chitosan	Dorzolamide	L-F	229 mg/g	[85]

L: Langmuir; F: Freundlich; L-F: Langmuir-Freundlich.

**Figure 9:**
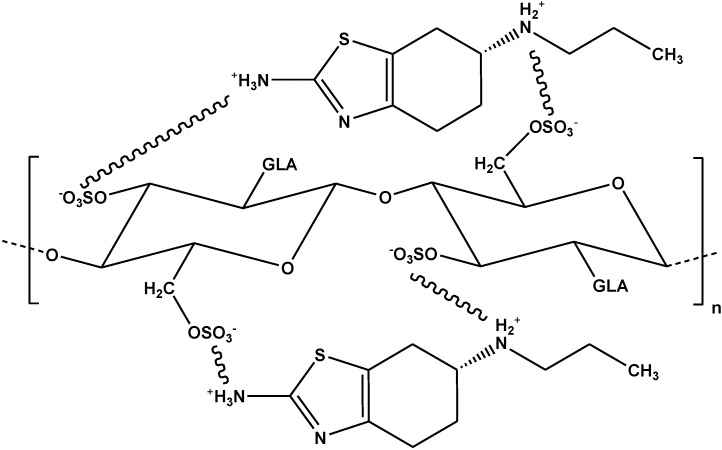
Interactions between pramipexole and CsSLF. Reprinted with permission from [84], Copyright © 2013 Elsevier.

In our previous study, we synthesized two chitosan modified derivatives which were cross-linked with glutaraldehyde and grafted with sulfonate groups (CsSLF) or *N*-(2-carboxybenzyl) groups (CsNCB) [84]. A pharmaceutical compound (pramipexole) was tested for adsorption experiments. After running pH-effect experiments, the optimum pH value was 10. After fitting to the Langmuir-Freundlich equation, the Q_m_ values were calculated as 337 and 307 mg/g at 25 °C for CsSLF and CsNCB, respectively. In this work, the most interesting aspect is the adsorption mechanism suggested for both materials. For example, in the case of CsSLF, the proposed mechanism is presented below (Figure 9).

**Figure 10:**
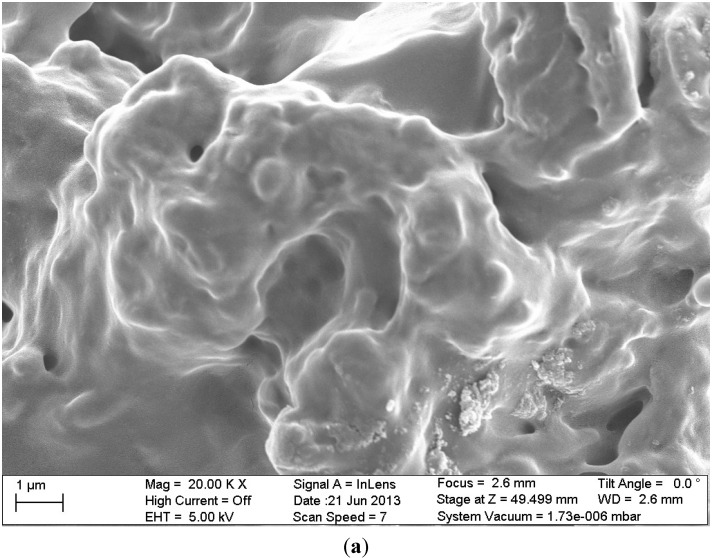

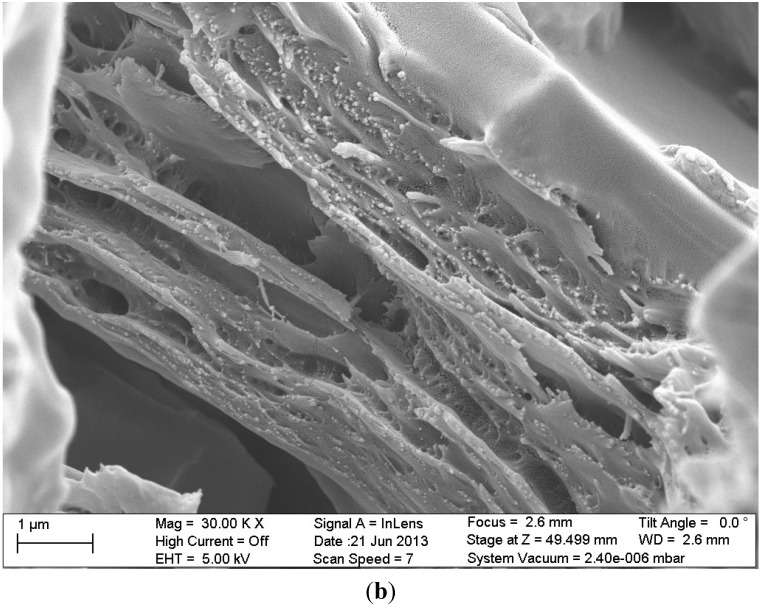
SEM images of the materials studied (**a**) chitosan cross-linked with glutaraldehyde and grafted with poly(acrylic acid) (CSA); (**b**) composite of cross-linked with glutaraldehyde chitosan which was grafted with poly(acrylic acid) and functionalized with graphite oxide (GO/CSA). Reprinted with permission from [85], Copyright © 2014 Elsevier.

**Figure 11:**
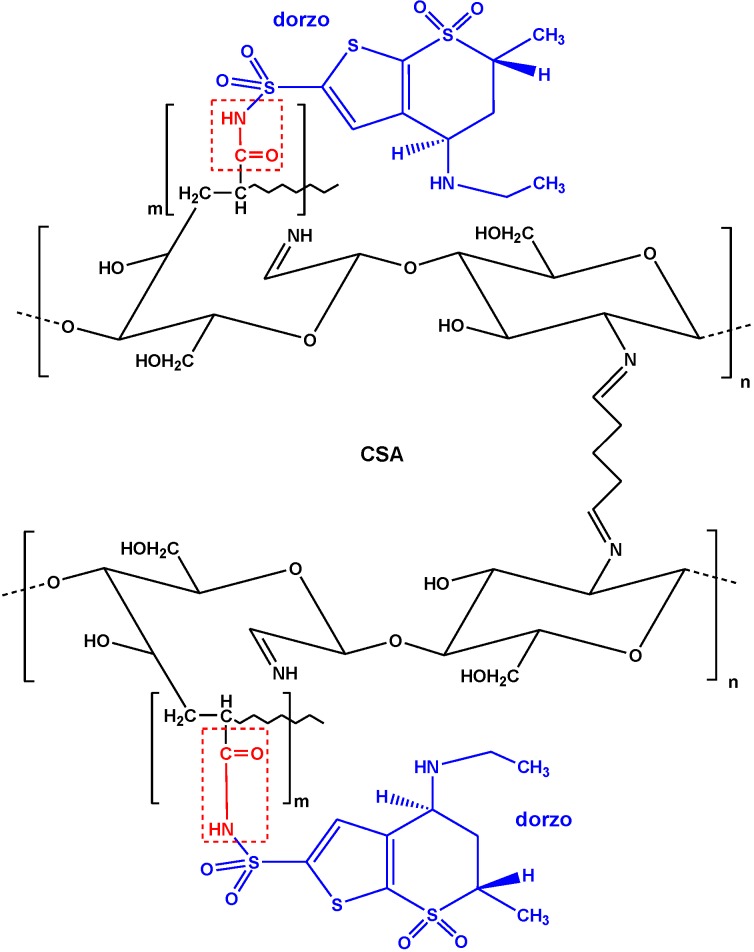
Interactions between CSA and dorzo. Reprinted with permission from [85], Copyright © 2014 Elsevier.

The same research team realized another modification in chitosan which was more complex. At first, chitosan was cross-linked with glutaraldehyde and grafted with poly(acrylic acid) to add extra carboxyl groups (denoted as CSA). Then, CSA was functionalized with graphite oxide to produce the final nanocomposite material (GO/CSA) [85]. The surface morphology of the prepared adsorbents is illustrated with SEM images in Figure 10. The latter was used as adsorbent material to remove a particular pharmaceutical compound (dorzolamide) that exists in wastewaters. The optimum pH value was 3. Q_m_ was calculated with L-F fitting model; for the composite GO/CSA was 334 mg/g, while for the origin materials GO and CSA were 175 and 229 mg/g, respectively.

Similarly, after characterization, the authors proposed an adsorption mechanism between dorzolamide (abbreviated as dorzo) and modified chitosan (CSA) (Figure 11).

## 5. Critical Comparisons

In adsorption technology, it is widely known that two adsorbent materials (even for the same pollutant) cannot be compared without maintaining the same experimental conditions. Some of the basic parameters which strongly influence the whole procedure are (i) the pH solution; (ii) contact time; (iii) initial pollutant’s concentration; (iv) temperature; (v) agitation speed; (vi) volume of adsorbate; (viii) ionic strength of solution; (ix) adsorbent’s dosage, *etc*. It is clear that if any of the aforementioned conditions vary, the experiment will not be the same and consequently no comparison will be correct. Having the above in mind, the only comparisons that can be realized are those for adsorbent/adsorbate systems of the same study.

Therefore, it is not correct to say that modified ball clay/chitosan composite [30] is a better adsorbent than β-cyclodextrin/chitosan magnetic nanocomposite [31], because the experimental conditions were not the same. The first material presented Q_m_ equal to 259.8 mg/g for MB adsorption, while the second presented a value of 2788 mg/g for the same dye. The same remark can be concluded for the removal of RB5 with adsorption onto chitosan grafted with polypropylene imine (Q_m_ = 6250 mg/g) [40], chitosan/graphite oxide composite (Q_m_ = 277 mg/g) [46] and magnetic chitosan/graphite oxide nanocomposite (Q_m_ = 391 mg/g) [47]. For those experiments, the adsorption conditions are completely different and therefore an accurate comparison cannot be made.

A similar approach can be takn for the case of metals. As it is clearly shown in Table 2, one of the most studied metals is Cu(II). Many researchers investigated the removal of this metal after modification of chitosan. For example, ethylene-1,2-diamine-6-deoxy-chitosan (Q_m_ = 41.6 mg/g) and ethylene-1,2-diamine-6-deoxy-*N*-phthaloylchitosan (Q_m_ = 31.3 mg/g) were synthesized in the literature [54]. However, this synthesis was undertaken with the addition of *N*-phthaloyl groups. The first modified derivative possessed enhanced ability for binding metal ions due to the presence of a flexible ethylenediamine substituent with two nitrogen basic centers in addition to the single bond NH_2_ at C-2 position. On the other hand, the grafting of *N*-phthaloyl groups renders the material more hydrophobic compared to the former material. Another study employing Cu(II) removal reports the synthesis of chitosan grafted with 4,4′-diformyl-α-ω-diphenoxy-ethane [60], presenting very small adsorption capacity (Q_m_ = 12 mg/g). It seems that the addition of 4,4′-diformyl-α-ω-diphenoxy-ethane via nucleophilic attack of the amino group of the chitosan on the carbonyl carbon of the aldehyde groups did not improve the adsorption ability of chitosan. Opposite observations were noticed after the adsorption tests on chloroacetic grafted chitosan and glycine grafted chitosan [65]. The grafting of chloroacetic acid on chitosan improved in higher percentages the adsorption capacity (Q_m_ = 175.12 mg/g) than did the addition of glycine groups (Q_m_ = 165.91 mg/g). The latter may be due to the structure of functional groups. Another study reported high capacities for modified derivatives which have been only cross-linked (either with glutaraldehyde (Q_m_ = 177.8 mg/g) or epichlorohydrin (Q_m_ = 150.4 mg/g)) and not grafted [66]. Obviously, the adsorption conditions were completely different and for this reason the materials prepared showed such high adsorption capacities.

Based on the above, when the grafting reaction of chitosan with various functional groups does not contain nitrogen carriers (for achieving a chelation reaction with metals), the adsorption capacity cannot be enhanced.

## 6. Concluding Remarks

Chitosan is a very promising adsorbent, which can be modified in many ways (grafting, cross-linking, functionalization for forming composites, *etc.*). The potential of chitosan is strong due to its origin product being chitin which can be found in abundance in marine media and especially in the exoskeleton of crustaceans, or cartilages of mollusks, cuticles of insects and cell walls of micro-organisms. However, one serious drawback of using chitosan as adsorbent is its swelling. For this reason, until now industrial use has been limited. However, some international companies purchase industrial grade chitosan for wastewater treatment (Qingdao Develop Chemistry Ltd in China, *etc.*). The recent modifications aim at strengthening the mechanical properties of chitosan in order for it to compete with activated carbon in commercial/industrial applications. A large volume of works has been published during the last three years, presenting results of chitosan-modified adsorbents for removal of various pollutants (dyes, metals/ions, others). It can be concluded that a limited number of works clearly presented novel modifications of the chitosan structure (finding new functional groups for grafting reactions or agents for cross-linking). The majority of works used already published modified materials and tested them for other/different types of pollutants. The latter may be due to the fact that the basic modifications have been completed and extensively published. Another possible reason may be the recent trend for publishing higher quantities of papers and not attempting to find some new modification. In any case, the potential of modified materials is still large and can be significantly developed in the future.

## Nomenclature of Dyes Reported in This Study

AG25: Acid green 25
AO7: Acid orange 7
AR18: Acid red 18
BBR250: Brilliant blue R250
BO16: Bezactive orange 16
CR: Congo red
MB: Methylene blue
RB19: Reactive blue 19
RB5: Reactive black 5
RR198: Reactive red 198
RR120: Reactive red 120
RR3BS: Remazol red 3BS
RY3RS: Reactive yellow 3RS
WASC: Weak acid scarlet
